# Report of Deaths in Buffalo for the Month of January, 1856

**Published:** 1856-03

**Authors:** C. L. Dayton

**Affiliations:** Health Physician


### ART. IX.—*Report of Deaths in Buffalo for the month*

| DISEASES. | Males. | Females. | Total. | AGE. Under 1 year. Male. | Under 1 year. Female. | 1 to 2 years. Male. | 1 to 2 years. Female. | 2—5 Male. | 2—5 Female. | 5—10 Male. | 5—10 Female. |
|---|---|---|---|---|---|---|---|---|---|---|---|
| Apoplexy, | 4 | 1 | 5 | | | | | | | | |
| Abscess, | 2 | | 2 | | | | | | | | |
| Atrophia, | 1 | 1 | 2 | 1 | | | 1 | | | | |
| Anasarca, | 1 | | 1 | | | | | | | | |
| Anæmia, | | 1 | 1 | | | | | | | | |
| Burned, | 2 | | 2 | | | 1 | | 1 | | | |
| Bronchitis, | 1 | | 1 | 1 | | | | | | | |
| Broken Leg, | 1 | | 1 | | | | | | | | |
| Consumption, | 11 | 4 | 15 | | | | | | | | |
| Convulsions, | 9 | 5 | 14 | 4 | 3 | | | 4 | | | |
| " Apoplectic, | 1 | 1 | 2 | | | | | | | | |
| Croup, | 2 | 2 | 4 | 1 | | 1 | 1 | | 1 | | |
| Cancer, | | 2 | 2 | | | | | | | | |
| Congestion of Lungs, | 2 | 1 | 3 | 1 | 1 | | | | | | |
| Child Bed, | | 2 | 2 | | | | | | | | |
| Delirium Tremens, | 3 | | 3 | | | | | | | | |
| Dissipation, | 3 | | 3 | | | | | | | | |
| Diarrhœa, | 2 | 1 | 3 | | | | | 2 | | | |
| Disease of Heart, | 1 | 1 | 2 | 1 | | | | | | | |
| Disease of Brain, | 1 | | 1 | | | | | | | | |
| Disease of Liver, | | 1 | 1 | | | | | | | | |
| Decline, | 1 | | 1 | 1 | | | | | | | |
| Debility, | | 1 | 1 | | | | | | | | |
| Enteritis, | | 1 | 1 | | | | | | | | |
| Erysipelas Phlegmonus, | | 1 | 1 | | | | | | | | |
| Exposure and Drunkenness, | | 1 | 1 | | | | | | | | |
| Effusion on the Brain, | 1 | | 1 | 1 | | | | | | | |
| Fever Typhoid, | 3 | 3 | 6 | | | | 2 | | | | |
| " Scarlet, | 3 | 2 | 5 | 1 | | 1 | | 1 | | | 2 |
| " Typhus, | 1 | | 1 | | | | | | | 1 | |
| " Nervosa, | 2 | 1 | 3 | | | | | | 1 | | |
| " Puerperal, | | 2 | 2 | | | | | | | | |
| Marasmus, | 2 | 1 | 3 | | | | | 1 | | 1 | |
| Meningitis, | 1 | | 1 | | | | | | | | |
| Hydrocephalus, | | 1 | 1 | | | | 1 | | | | |
| Old Age, | 1 | 1 | 2 | | | | | | | | |
| Pneumonia, | 5 | 3 | 8 | | | 1 | | | | 2 | |
| Peritonitis, Puerperal, | | 1 | 1 | | | | | | | | |
| Still-born, | 2 | 1 | 3 | 2 | 1 | | | | | | |
| Scrofula, | 1 | | 1 | 1 | | | | | | | |
| Scirrus Pylori, | | 1 | 1 | | | | | | | | |
| Spasms, | 1 | | 1 | 1 | | | | | | | |
| Teething, | 2 | | 2 | 1 | | 1 | | | | | |
| Unknown, | 1 | 2 | 3 | 1 | 2 | | | | | | |
| Whooping Cough, | 1 | | 1 | | | 1 | | | | | |
| Totals, | 75 | 46 | 121 | 18 | 7 | 6 | 5 | 9 | 2 | 4 | 2 |

NATIVITIES.—Americans, (whites) 48, (colored) 4; total 52. German, 24. English, 2.

*of January,* 1856. By C. L. DAYTON, M. D., Health Physician.

| AGE. 10—15 | | 15—20 | | 20—25 | | 25—30 | | 30—35 | | 35—40 | | 40—45 | | 45—50 | | 50—60 | | 60—70 | | 70—80 | | 80—90 | | 90—100 | | 100 and upward. | | Not given. | |
|---|---|---|---|---|---|---|---|---|---|---|---|---|---|---|---|---|---|---|---|---|---|---|---|---|---|---|---|---|---|
| Male. | Female. | Male. | Female. | Male. | Female. | Male. | Female. | Male. | Female. | Male. | Female. | Male. | Female. | Male. | Female. | Male. | Female. | Male. | Female. | Male. | Female. | Male. | Female. | Male. | Female. | Male. | Female. | Male. | Female. |
| .. | .. | .. | .. | .. | .. | .. | .. | .. | .. | .. | .. | .. | .. | .. | .. | 3 | 1 | 1 | .. | .. | .. | .. | .. | .. | .. | .. | .. | .. | .. |
| .. | .. | .. | .. | .. | .. | .. | .. | .. | .. | .. | .. | 2 | .. | .. | .. | .. | .. | .. | .. | .. | .. | .. | .. | .. | .. | .. | .. | .. | .. |
| .. | .. | .. | .. | .. | .. | .. | .. | .. | .. | .. | .. | .. | .. | .. | .. | .. | .. | .. | .. | .. | .. | .. | .. | .. | .. | .. | .. | .. | .. |
| .. | .. | 1 | .. | .. | .. | .. | .. | .. | .. | .. | .. | .. | .. | .. | .. | .. | .. | .. | .. | .. | .. | .. | .. | .. | .. | .. | .. | .. | .. |
| .. | .. | .. | 1 | .. | .. | .. | .. | .. | .. | .. | .. | .. | .. | .. | .. | .. | .. | .. | .. | .. | .. | .. | .. | .. | .. | .. | .. | .. | .. |
| .. | .. | .. | .. | .. | .. | .. | .. | .. | .. | .. | .. | .. | .. | .. | .. | .. | .. | .. | .. | .. | .. | .. | .. | .. | .. | .. | .. | .. | .. |
| .. | .. | .. | .. | .. | .. | .. | .. | .. | .. | .. | .. | .. | .. | .. | .. | .. | .. | .. | .. | .. | .. | .. | .. | .. | .. | .. | .. | .. | .. |
| .. | .. | .. | .. | .. | .. | .. | .. | .. | .. | .. | .. | 1 | .. | .. | .. | .. | .. | .. | .. | .. | .. | .. | .. | .. | .. | .. | .. | .. | .. |
| 1 | .. | 1 | 1 | 1 | .. | .. | 1 | 3 | 1 | .. | .. | 1 | 1 | 2 | .. | 1 | .. | 1 | .. | .. | .. | .. | .. | .. | .. | .. | .. | .. | .. |
| .. | 1 | .. | .. | .. | .. | .. | .. | .. | 1 | .. | .. | .. | .. | 1 | .. | .. | .. | .. | .. | .. | .. | .. | .. | .. | .. | .. | .. | .. | .. |
| .. | .. | .. | .. | .. | .. | .. | .. | .. | 1 | .. | .. | 1 | .. | .. | .. | .. | .. | .. | .. | .. | .. | .. | .. | .. | .. | .. | .. | .. | .. |
| .. | .. | .. | .. | .. | .. | .. | .. | .. | .. | .. | .. | .. | .. | .. | .. | .. | .. | .. | .. | .. | .. | .. | .. | .. | .. | .. | .. | .. | .. |
| .. | .. | .. | .. | .. | .. | .. | .. | .. | .. | .. | 1 | .. | .. | .. | .. | .. | .. | .. | 1 | .. | .. | .. | .. | .. | .. | .. | .. | .. | .. |
| .. | .. | .. | .. | .. | .. | .. | .. | .. | .. | .. | .. | .. | .. | .. | .. | 1 | .. | .. | .. | .. | .. | .. | .. | .. | .. | .. | .. | .. | .. |
| .. | .. | .. | .. | .. | 1 | .. | 1 | .. | .. | .. | .. | .. | .. | .. | .. | .. | .. | .. | .. | .. | .. | .. | .. | .. | .. | .. | .. | .. | .. |
| .. | .. | .. | .. | 1 | .. | .. | .. | 1 | .. | .. | .. | .. | .. | 1 | .. | .. | .. | .. | .. | .. | .. | .. | .. | .. | .. | .. | .. | .. | .. |
| .. | .. | .. | .. | 1 | .. | .. | .. | .. | .. | .. | .. | 1 | .. | .. | .. | .. | .. | 1 | .. | .. | .. | .. | .. | .. | .. | .. | .. | .. | .. |
| .. | .. | .. | .. | .. | .. | .. | 1 | .. | .. | .. | .. | .. | .. | .. | .. | .. | .. | .. | .. | .. | .. | .. | .. | .. | .. | .. | .. | .. | .. |
| .. | .. | .. | .. | .. | .. | .. | .. | .. | .. | .. | .. | .. | .. | .. | .. | .. | .. | .. | .. | .. | 1 | .. | .. | .. | .. | .. | .. | .. | .. |
| .. | .. | .. | .. | .. | .. | .. | .. | .. | .. | .. | .. | 1 | .. | .. | .. | .. | .. | .. | .. | .. | .. | .. | .. | .. | .. | .. | .. | .. | .. |
| .. | .. | .. | .. | .. | .. | .. | .. | .. | .. | .. | .. | .. | .. | .. | .. | .. | 1 | .. | .. | .. | .. | .. | .. | .. | .. | .. | .. | .. | .. |
| .. | .. | .. | .. | .. | .. | .. | .. | .. | .. | .. | .. | .. | .. | .. | .. | .. | .. | .. | .. | .. | .. | .. | .. | .. | .. | .. | .. | .. | .. |
| .. | .. | .. | .. | .. | .. | .. | 1 | .. | .. | .. | .. | .. | .. | .. | .. | .. | .. | .. | .. | .. | .. | .. | .. | .. | .. | .. | .. | .. | .. |
| .. | .. | .. | 1 | .. | .. | .. | .. | .. | .. | .. | .. | .. | .. | .. | .. | .. | .. | .. | .. | .. | .. | .. | .. | .. | .. | .. | .. | .. | .. |
| .. | .. | .. | .. | .. | .. | .. | .. | .. | .. | .. | .. | .. | .. | .. | .. | .. | 1 | .. | .. | .. | .. | .. | .. | .. | .. | .. | .. | .. | .. |
| .. | .. | .. | .. | .. | 1 | .. | .. | .. | .. | .. | .. | .. | .. | .. | .. | .. | .. | .. | .. | .. | .. | .. | .. | .. | .. | .. | .. | .. | .. |
| .. | .. | .. | .. | .. | .. | .. | .. | .. | .. | .. | .. | .. | .. | .. | .. | .. | .. | .. | .. | .. | .. | .. | .. | .. | .. | .. | .. | .. | .. |
| 2 | 1 | 1 | .. | .. | .. | .. | .. | .. | .. | .. | .. | .. | .. | .. | .. | .. | .. | .. | .. | .. | .. | .. | .. | .. | .. | .. | .. | .. | .. |
| .. | .. | .. | .. | .. | .. | .. | .. | .. | .. | .. | .. | .. | .. | .. | .. | .. | .. | .. | .. | .. | .. | .. | .. | .. | .. | .. | .. | .. | .. |
| .. | .. | .. | .. | .. | .. | .. | .. | .. | .. | .. | .. | .. | .. | .. | .. | .. | .. | .. | .. | .. | .. | .. | .. | .. | .. | .. | .. | .. | .. |
| .. | .. | 2 | .. | .. | .. | .. | .. | .. | .. | .. | .. | .. | .. | .. | .. | .. | .. | .. | .. | .. | .. | .. | .. | .. | .. | .. | .. | .. | .. |
| .. | .. | .. | .. | .. | 2 | .. | .. | .. | .. | .. | .. | .. | .. | .. | .. | .. | .. | .. | .. | .. | .. | .. | .. | .. | .. | .. | .. | .. | .. |
| .. | .. | .. | .. | .. | .. | .. | .. | .. | .. | .. | .. | .. | .. | .. | .. | .. | .. | .. | .. | .. | .. | .. | .. | .. | 1 | .. | .. | .. | .. |
| .. | .. | .. | .. | 1 | .. | .. | .. | .. | .. | .. | .. | .. | .. | .. | .. | .. | .. | .. | .. | .. | .. | .. | .. | .. | .. | .. | .. | .. | .. |
| .. | .. | .. | .. | .. | .. | .. | .. | .. | .. | .. | .. | .. | .. | .. | .. | .. | .. | .. | .. | .. | .. | .. | .. | .. | .. | .. | .. | .. | .. |
| .. | .. | .. | .. | .. | .. | .. | .. | .. | .. | .. | .. | .. | .. | .. | .. | .. | .. | 1 | .. | .. | 1 | .. | .. | .. | .. | .. | .. | .. | .. |
| .. | .. | .. | .. | .. | 1 | 1 | 1 | .. | .. | .. | .. | .. | .. | 1 | .. | .. | .. | .. | .. | .. | 1 | .. | .. | .. | .. | .. | .. | .. | .. |
| .. | .. | .. | .. | .. | .. | .. | .. | .. | 1 | .. | .. | .. | .. | .. | .. | .. | .. | .. | .. | .. | .. | .. | .. | .. | .. | .. | .. | .. | .. |
| .. | .. | .. | .. | .. | .. | .. | .. | .. | .. | .. | .. | .. | .. | .. | .. | .. | .. | .. | .. | .. | .. | .. | .. | .. | .. | .. | .. | .. | .. |
| .. | .. | .. | .. | .. | .. | .. | .. | .. | .. | .. | .. | .. | .. | .. | .. | .. | .. | .. | .. | .. | .. | .. | .. | .. | .. | .. | .. | .. | .. |
| .. | .. | .. | .. | .. | .. | .. | .. | .. | .. | .. | .. | .. | .. | .. | .. | .. | .. | .. | 1 | .. | .. | .. | .. | .. | .. | .. | .. | .. | .. |
| .. | .. | .. | .. | .. | .. | .. | .. | .. | .. | .. | .. | .. | .. | .. | .. | .. | .. | .. | .. | .. | .. | .. | .. | .. | .. | .. | .. | .. | .. |
| .. | .. | .. | .. | .. | .. | .. | .. | .. | .. | .. | .. | .. | .. | .. | .. | .. | .. | .. | .. | .. | .. | .. | .. | .. | .. | .. | .. | .. | .. |
| .. | .. | .. | .. | .. | .. | .. | .. | .. | .. | .. | .. | .. | .. | .. | .. | .. | .. | .. | .. | .. | .. | .. | .. | .. | .. | .. | .. | .. | .. |
| .. | .. | .. | .. | .. | .. | .. | .. | .. | .. | .. | .. | .. | .. | .. | .. | .. | .. | .. | .. | .. | .. | .. | .. | .. | .. | .. | .. | .. | .. |
| .. | .. | .. | .. | .. | .. | .. | .. | .. | .. | .. | .. | .. | .. | .. | .. | .. | .. | .. | .. | .. | .. | .. | .. | .. | .. | .. | .. | .. | .. |
| .. | .. | .. | .. | .. | .. | .. | .. | .. | .. | .. | .. | .. | .. | .. | .. | .. | .. | .. | .. | .. | .. | .. | .. | .. | .. | .. | .. | .. | .. |
| .. | .. | .. | .. | .. | .. | .. | .. | .. | .. | .. | .. | .. | .. | .. | .. | .. | .. | .. | .. | .. | .. | .. | .. | .. | .. | .. | .. | .. | .. |
| .. | .. | .. | .. | .. | .. | .. | .. | .. | .. | .. | .. | .. | .. | .. | .. | .. | .. | .. | .. | .. | .. | .. | .. | .. | .. | .. | .. | .. | .. |
| .. | .. | .. | .. | .. | .. | .. | .. | .. | .. | .. | .. | .. | .. | .. | .. | .. | .. | .. | .. | .. | .. | .. | .. | .. | .. | .. | .. | .. | .. |
| .. | .. | .. | .. | .. | .. | .. | .. | .. | .. | .. | .. | .. | .. | .. | .. | .. | .. | .. | .. | .. | .. | .. | .. | .. | .. | .. | .. | .. | .. |
| .. | .. | .. | .. | .. | .. | .. | .. | .. | .. | .. | .. | .. | .. | .. | .. | .. | .. | .. | .. | .. | .. | .. | .. | .. | .. | .. | .. | .. | .. |
| 3 | 2 | 5 | 3 | 4 | 5 | 1 | 5 | 4 | 4 | 0 | 1 | 7 | 1 | 5 | 0 | 5 | 3 | 4 | 2 | 0 | 3 | 0 | 0 | 0 | 1 | 0 | 0 | 0 | 0 |

Irish, 19. Swiss, 1. French, 6. Canadian, 2. Country not given, 15.—Total, 121.

